# User Re-Identification via Confusion of the Contrastive Distillation Network and Attention Mechanism

**DOI:** 10.3390/s23198170

**Published:** 2023-09-29

**Authors:** Mingming Zhang, Bin Wang, Sulei Zhu, Xiaoping Zhou, Tao Yang, Xi Zhai

**Affiliations:** 1 College of Information, Mechanical and Electrical Engineering, Shanghai Normal University, Shanghai 200234, China; mingmingzhang3064@163.com (M.Z.); zhouxp@shnu.edu.cn (X.Z.); 2 Shanghai Urban and Rural Construction and Traffic Development Research Institute, Shanghai 200234, China; yangtaocoolboy@163.com (T.Y.); jessi_zx28@163.com (X.Z.)

**Keywords:** user re-identification, contrastive distillation network, transformer

## Abstract

With the rise of social networks, more and more users share their location on social networks. This gives us a new perspective on the study of user movement patterns. In this paper, we solve the trajectory re-identification task by identifying human movement patterns and then linking unknown trajectories to the user who generated them. Existing solutions generally focus on the location point and the location point information, or a single trajectory, and few studies pay attention to the information between the trajectory and the trajectory. For this reason, in this paper, we propose a new model based on a contrastive distillation network, which uses a contrastive distillation model and attention mechanisms to capture latent semantic information for trajectory sequences and focuses on common key information between pairs of trajectories. Combined with the trajectory library composed of historical trajectories, it not only reduces the number of candidate trajectories but also improves the accuracy of trajectory re-identification. Our extensive experiments on three real-world location-based social network (LBSN) datasets show that our method outperforms existing methods.

## 1. Introduction

With the popularization of mobile devices and the development of mobile computing technology, the amount of spatio-temporal trajectory data has shown explosive growth. Spatio-temporal trajectory data record the behavior trajectory of individuals in space and time, such as GPS positioning data of mobile devices, check-in data on social networks, data for card payments in public transportation systems, etc. Analyzing these data will help us to dig into human movement patterns and behavior patterns, predict and model human behavior, and develop intelligent business models to improve user experience. It can also provide effective solutions for urban intelligent transportation [1,2], personalized interest point recommendation [3,4,5], friend recommendation, character relationship extraction [6], location prediction [7,8], criminal detection [9,10,11], public safety [12], and other issues.

The user re-identification task is basic in the analysis of human movement behavior. It links unknown trajectories to the corresponding user to achieve the purpose of re-identification. User re-identification can be applied to many fields of trajectory data mining. For example, applications such as shared bicycles collect a large amount of user behavior trajectory information in the background, but this user ID information is hidden. Social networks such as Gowalla, Weeplaces, Foursquare, and Facebook Places [13] collect detailed information such as restaurants, shopping centers, and hotels that are visited by users. When users access an application, they generate mobile data in the background. When the accumulated trajectory data are sufficient, these mobile data can be connected to form a complete mobile trajectory sequence. Through these complete mobile trajectory sequences, more human mobile mode information can be tapped. Commonly used human trajectory modeling methods such as the Markov chain (MC) [14] and recurrent neural network (RNN) are based on historical check-ins modeling human liquidity.

Solving the problem of user re-identification can involve adopting general classification methods, such as longest common subsequence (LCSS), edit distance on real sequence (EDR), dynamic time warping (DTW), and other methods based on trajectory similarity. Another possibility is creating a solution based on a deep neural network model. Ref. [15] first used deep learning models to deal with trajectory–user link problems. The embedding method is used to embed the user-generated position into the vector space, and the recurrent neural network model is used to model the trajectory information. Because RNN can input a variable-length trajectory sequence and capture the long-term dependence of the position, the result achieves good performance. The TULVAE [16] model improves on the former model by learning high-level semantic information regarding trajectories and using semi-supervised learning to improve linking capabilities. Following this, ref. [17] proposed an end-to-end attentional circulation neural learning framework (TULAR). Despite the effectiveness of the current methods, they struggle to make accurate predictions at a high level, for the following reasons: (1) check-in data are sparse. User check-in data generally do not have a fixed frequency and rules, which can lead to noise or abnormal values. These problems have presented huge challenges to re-identification tasks. (2) The existing models struggle to distinguish between similar embedding vectors and cannot capture the dependency of the trajectory sequences. (3) Most models lack effective use of historical trajectories. Establishing how to effectively use historical trajectory information is also a major challenge.

To solve the above challenges, we propose a model of a contrastive distillation network. In this model, we use the Word2vec [18] method for sparse social network datasets to convert the trajectory data into the form of word vectors, each of which corresponds to the characteristics of a location point. To better distinguish between similar vectors and capture trajectory dependencies, we propose a network model based on a contrastive framework, using RNN and Transformer models to learn the differences between trajectory pairs, and adding a global attention mechanism to learn the correlation of trajectories. For the use of historical trajectories, we establish a user-to-location mapping relationship, called the trajectory–user library. When given an unknown trajectory, we go to the trajectory–user library to query the corresponding candidate user, and then send the unknown trajectory and the corresponding candidate user’s trajectory pair into our model to calculate the similarity score. Finally, we use a statistical-based method to determine user IDs with unknown trajectories. The main contributions of this work are as follows:

- We propose a contrastive distillation network model to solve the problem of user re-identification. This model combines the advantages of the distillation model and the contrastive learning framework. To the best of our knowledge, our model is the first model to combine the user–trajectory library with the contrastive distillation network, which greatly improves the versatility and rationality of the task.
- We design a supervised contrastive learning framework based on an RNN trajectory encoder and Transformer trajectory encoder to learn the latent semantic features of trajectory sequences, combined with the attention mechanism, focusing on learning the common key information between trajectory pairs.
- We conduct extensive experiments on three real public datasets. Experimental results verify the effectiveness of the model and outperform the existing methods.

The rest of this article is structured as follows. In Section 2, we introduce the work related to the re- identification tasks of trajectory. After that, a statement of a short trajectory re-identification is given in Section 3. Section 4 describes the details and frameworks of the entire network of our model. Section 5 introduces the evaluation methods and experimental results, and the paper is summarized in Section 6.

## 2. Related Work

With the popularity of social networking software and the advancement of location acquisition technology, trajectory data mining is becoming an increasingly important research topic. These trajectory data represent the mobility of human behavior, providing unprecedented information for understanding patterns of human behavior and contributing to the widespread use of location-based social networks [19], intelligent transportation systems, and urban computing [20]. Aiming at the trajectory data, the problems of trajectory pattern mining, trajectory uncertainty, outlier detection, trajectory classification, and personalized interest recommendation are proposed. User re-identification is also an important task in trajectory data mining, and its main task is to associate the trajectory with the user who generated the trajectory. Research on social network-based user mobility is becoming the focus of research.

The common ways to solve trajectory classification is to use probability graph models, such as dynamic Bayesian networks (DBN), hidden Markov model (HMM), and conditional random fields (CRF), but these methods struggle to capture the dependencies and spatiotemporal characteristics between trajectory sequences. It can also be solved using classification methods commonly used in machine learning, such as SVM [21], KNN [22], and random forest [23]. In recent years, researchers have begun to use deep learning methods to study the problem of trajectory re-identification. Ref. [15] first adopted deep learning methods to solve the problem of user trajectory linking. In subsequent research, ref. [16] used variational autoencoders to improve the TULER model and proposed the TULVAE model framework. Ref. [24] proposed the AdattTUL model to dynamically capture the complex relationship of user check-in from trajectory data. Ref. [25] proposed the DeepTUL model, which not only combines multiple characteristics of user mobility to model high-order complex movement patterns but also learns from historical trajectories to capture the multi-periodicity of user mobility and alleviate the problem of data sparsity. Ref. [17] proposed an end-to-end attentional neural learning framework (TULAR). Ref. [26] proposed a new mutual distillation learning network (MainTUL) to solve the TUL problem of sparse check-in mobile data. From the perspective of calculating trajectory similarity, traditional methods include longest common subsequence (LCSS) [27], editing distance on real sequence (EDR) [28], dynamic time warp (DTW) [29], etc., but these research methods focus more on improving the efficiency of trajectory similarity calculation. Based on deep semantic similarity calculation, it is possible to learn the internal relationships of trajectories. In this work, semantic trajectory similarity is calculated based on our contrastive distillation network, and the differences between pairs of trajectories are learned, capturing long-term dependencies between locations. In addition, for the representation of user tracks, we add a global attention mechanism that allows the model to learn the correlation between trajectories.

## 3. Preliminaries

### 3.1. Problem Definition

Let $T_{u_{i}}=\{l_{i1},l_{i2},l_{i3},\ldots ,l_{in}\}$ denote a trajectory generated by the user $u_{i}$ in a period of time, where $l_{ij}\left(j\in \{1,2,\ldots ,n\}\right)$ is a location at time $t_{j}$ for the user $u_{i}$. For spatio-temporal trajectory information $T_{u_{p}}=\{l_{p1},l_{p2},l_{p3},\ldots ,l_{pn}\}$, we know that $u_{p}$ generated it, meaning that this situation is labeled as *identified*. But, for the trajectory $T_{u_{q}}=\{l_{q1},l_{q2},l_{q3},\ldots ,l_{qn}\}$, we know the trajectory data but do not know who generated them, meaning that this situation is labeled as *unidentified*. The re-identification task involves finding the user who generated the trajectory $T_{u_{q}}$ and converting its status to *identified*.

### 3.2. Trajectory Pre-Processing

To facilitate our modeling, we need to divide the user-generating sequence of long-term trajectories into continuous sub-trajectories (e.g., 6 h, one day, or one week) that follow the lifestyle of human behavior. The purpose is to study the characteristics of human space–time motion and provide richer knowledge for semantic trajectory analysis such as trajectory data mining. The social network dataset we choose comprises a sequence of sample points containing information such as latitude and longitude, time points, user IDs, etc. Due to the sparseness of the social network dataset, we need to preprocess it to a certain extent. Less frequent check-ins by users mean that those spots are visited by fewer users. This affects the reliability and accuracy of the results. Deleting some less frequent data points can make the data more representative. In this way, the data can better reflect the overall user behavior and geographical distribution, and make the data more generalizable. We adopted the method in [15] to improve the reliability and generalization of our dataset.

### 3.3. Data Construction

Our model is built on a contrastive learning network. Contrastive learning network models are usually composed of two identical or similar networks, and their training data generally exist in the form of a sample pair, so we need to build positive and negative samples to train the model. The input to the model is of the form (trajectory A, trajectory B, label), and if the label is 1, we can establish that trajectory A and trajectory B are from the same user. If the label is 0, we can establish that trajectory A and trajectory B are from different users.

## 4. Proposed Method

**Figure 1:**
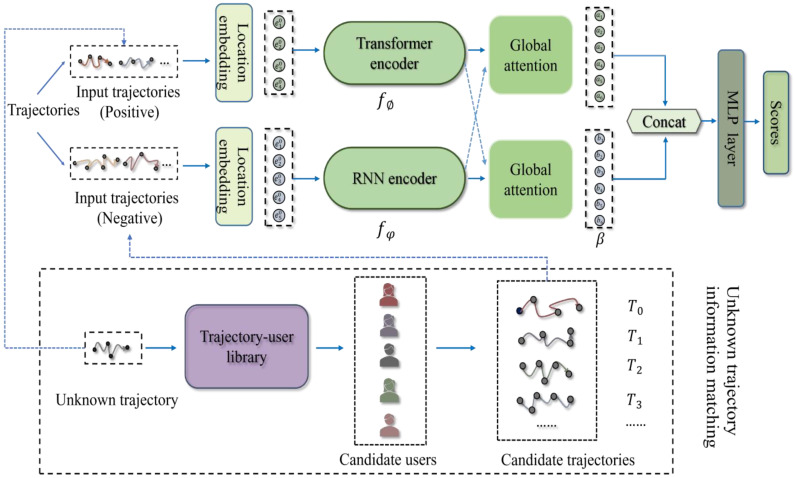
Our framework of user re-identification is mainly composed of a trajectory embedding layer, contrastive distillation encoder layer, global attention layer, output layer, and unknown trajectory information matching.

In this section, we will describe our proposed model of the contrastive distillation network, which is shown in Figure 1. It should be noted here that our contrastive distillation network does not strictly distinguish between teacher and student networks like [30], but learns from scratch. We use the trajectory embedding layer to turn the trajectory pair into the form of a feature vector, and then input the feature vector representation of the trajectory pair into the RNN encoder and Transformer encoder layer for learning, and the output of the RNN encoder and Transformer encoder layer is concatenated using the attention layer to learn the connection of trajectory pairs and obtain the context of the laws of human activity with higher semantics. Finally, we will perform feature fusion by comparing the features learned by the distillation model to obtain better model performance. When identifying an unknown trajectory, we need to calculate the similarity between it and the candidate trajectory in the trajectory user library to complete the re-identification task—see Section 4.5 for details.

### 4.1. Trajectory Embedding Layer

Social network datasets contain information such as latitude and longitude, time, user ID, location number, etc., which are complex and heterogeneous and cannot be directly entered into the model. We use a fixed vector of dimensions to represent each location point, but if we use common one-hot encoding, there is a dimensional curse. This is because there are usually tens of thousands of location points in the dataset so one-hot encoding will have tens of thousands of dimensions, which is obviously not feasible. In addition, the use of fixed-dimensional vectors can alleviate the sparsity of social network data. Specifically, we use Word2vec in natural language processing to learn the embedding expression of each location point, and the calculation formula of the trajectory embedding layer is as follows:

(1) $$V_{i}=tanh(W*L_{i}+b)$$

where $V_{i}$, W, and b are the learnable parameters of the trajectory embedding layer, and $L_{i}$ represents the ID of the location point.

### 4.2. Contrastive Distillation Encoder Layer

The contrastive distillation encoding layer is built based on the contrastive learning framework and two different models in the distillation network. In order to make the contrastive distillation encoding layer learn the differences between trajectories, we use two different basic models, RNN and Transformer. RNN is a common model for processing sequence data, and Transformer can process both sequence data and non-sequence data.

#### 4.2.1. RNN Encoder

RNN is an efficient architecture for handling variable-length sequences. Due to the sparsity of check-in trajectories, we used two variants of RNN (long short-term memory network (LSTM) [31] and gate recurrent unit (GRU) [32]) as encoders for $f_{\emptyset }$ to process the input trajectories. The architecture of LSTM consists of a memory cell and three gates, namely an input gate i, output gate o, and forget gate f. The specific formula is as follows:

(2) $$i_{t}=\sigma (W_{i}x_{t}+U_{i}h_{t-1}+V_{i}c_{t-1}+b_{i})$$

(3) $$o_{t}=\sigma (W_{o}x_{t}+U_{o}h_{t-1}+V_{o}c_{t-1}+b_{o})$$

(4) $$f_{t}=\sigma (W_{f}x_{t}+U_{f}h_{t-1}+V_{f}c_{t-1}+b_{f})$$

where $i_{t}$, $o_{t}$, and $f_{t}$ represent the input gate, output gate, and forget gate, respectively. σ represents the activation function. $W_{i}$, $U_{i}$, $V_{i}$, and $b_{i}$ represent learnable parameters of the input gate. $W_{o}$, $U_{o}$, $V_{o}$*,* and $b_{o}$ represent learnable parameters of the output gate. $W_{f}$, $U_{f}$, $V_{f}$, and $b_{f}$ represent learnable parameters of the forget gate, and $x_{t}$ represents the output vector of the trajectory embedding layer. $h_{t-1}$ represents the previous moment hidden state, $c_{t}$ represents the current cell state, $c_{t-1}$ represents the previous moment cell state, and $c_{t}$ is updated by

(5) $$c_{t}=f_{t}⊙c_{t-1}+i_{t}⊙tanh(W_{c}x_{t}+U_{c}h_{t-1}+b_{c})$$

The state $h_{t}$ is then updated by

(6) $$h_{t}=o_{t}⊙tanh(c_{t})$$

where $tanh\left(\cdot \right)$ refers to the hyperbolic tangent function, and ⊙ is the entry-wise product. $W_{c}$, $U_{c}$, and $b_{c}$ represent learnable parameters of the cell state, $x_{t}$ represents the input for the current time step, and $h_{t}$ represents the current hidden state.

Similar to LSTM, the GRU model has two gates, namely the update gate and the reset gate:

(7) $$z_{t}=\sigma (W_{z}x_{t}+U_{z}h_{t-1})$$

(8) $$r_{t}=\sigma (W_{r}x_{t}+U_{r}h_{t-1})$$

We update the state of $h_{t}$ by a linear interpolation between the last state $h_{t-1}$ and the state $\tilde{h_{t}}$ as

(9) $$h_{t}=\left(1-z_{t}\right)h_{t-1}+z_{t}\tilde{h_{t}}$$

The update formula of $\tilde{h_{t}}$ is as follows:

(10) $$\tilde{h_{t}}=tanh\left(Wx_{t}+U\left(r_{t}⊙h_{t-1}\right)\right)$$

where W is the weight matrix of the $x_{t}$, $x_{t}$ is the input of the current time step, U is the weight matrix of the hidden state of the previous time step, $z_{t}$ represents the update gate, $r_{t}$ represents the reset gate, ⊙ is the entry-wise product, and $h_{t-1}$ is the hidden state of the previous time step.

#### 4.2.2. Transformer Encoder

In [33], Google proposed the Transformer model, which uses a self-attention structure to replace the RNN network structure commonly used in NLP tasks [34] and other tasks [35]. Compared with the RNN network structure, its biggest advantage is that it can be computed in parallel. The Transformer model consists of positional embeddings, an attention module, a feed forward network, and a residual regularization module. The traditional convolutional neural networks (CNN) [36] and RNN structure are abandoned in the Transformer, and the entire network structure is composed entirely of the attention mechanism, and the addition of positional embeddings allows the model to learn the sequence of sequences. Based on the attention mechanism, the transformer model can mine the nonlinear feature relationship between trajectory time information and spatial position. In the transformer model, the attention module is composed of a self-attention mechanism and a multi-head attention mechanism.

For the self-attention machine, we need three matrices: matrix Q, matrix K, and matrix V as inputs, where matrix Q and matrix $K^{T}$ are dot products, and then we multiply by 1/$\sqrt{d_{k}}$, perform the softmax function, and finally multiply by the matrix V. The specific formula is as follows:

(11) $$Attention(Q,K,V)=softmax(\frac{QK^{T}}{\sqrt{d_{k}}})V$$

where $d_{k}$ is the dimension of the vector k in the formula, and the reason for multiplying by 1/$\sqrt{d_{k}}$ is twofold. Firstly, it is performed to increase the value of $QK^{T}$ moderately. Secondly, it is used to prevent the gradient disappearance in backpropagation caused by excessively large values when the dimension is too large.

Another type of multi-head attention allows the model to collectively focus on information that represents subspaces from different locations. Thus, the potential features of the trajectory sequence can be co-generated by different representation subspaces to improve the performance of the attention layer, as follows:

(12) $$MultiHead(Q,K,V)=Concat(head_{1},\ldots head_{n})W^{O}$$

where $head_{i}=Attention_{i}\left(QW_{i}^{Q},KW_{i}^{K},VW_{i}^{V}\right)$, and $head_{i}$ represents the *i*-th head of the multi-head attention mechanism. $W^{Q}$, $W^{K}$, and $W^{V}$ are learnable parameter matrices, and $W^{O}$ is an output fully connected layer that combines n heads.

After global attention, there is a residual connection and regularization module layer to solve the problem of gradient vanishing and the degradation of the weight matrix, as follows:

(13) LayerNorm(x+Feedforward(x))

Connecting a fully connected layer after the residual module, the fully connected feedforward network consists of two linear transformations with the ReLU activation function

(14) $$Feedforward(x)=max(0,xW_{1}+b_{1})W_{2}+b_{2}$$

Due to the sparse feature of location-based social network [19] datasets, the RNN network will forget some important location information of the user, and it is difficult to fully mine the user’s check-in behavior pattern through time series. The attention mechanism of the Transformer network can focus on the entire trajectory sequence. In our contrastive distillation model, both the Transformer model and RNN model are used. Two encoders with different performances will make our entire model more generalizable and decoupled better.

### 4.3. Global Attention Layer

The main principle of the attention mechanism [37] is to find key information from a large amount of information. In recent years, the combination of RNN and the attention mechanism has been very common, such as TULAR [16] and Deep TUL [25], but these models only focus on the connections between location points. In our paper, in order to enable our comparison framework to find key information from trajectory to trajectory, we also combine the comparison framework and attention mechanism, introducing a global attention mechanism. We set the input of the global attention mechanism as

(15) $$h_{t}={\bar{output}}_{model}$$

(16) $${\bar{h}}_{s}=output_{model}$$

where ${\bar{output}}_{model}$ represents the trajectory average feature representation of the Transformer encoder or RNN encoder’s output, and $output_{model}$ represents the trajectory feature representation of the Transformer encoder or RNN encoder’s output. model represents the Transformer encoder or RNN encoder. The global attention score is calculated as follows:

(17) $$\alpha_{t}=\frac{exp\left(score\left(h_{t},{\bar{h}}_{s}\right)\right)}{\sum_{s^{\prime }}exp\left(score\left(h_{t},{\bar{h}}_{s^{\prime }}\right)\right)}$$

(18) $$score\left(h_{t},{\bar{h}}_{s}\right)=\left\{\begin{array}{cc} h_{t}^{⊤}{\bar{h}}_{s} & dot\\ h_{t}^{⊤}W{\bar{h}}_{s} & general\\ v^{⊤}\tanh\left(W\left[h_{t};{\bar{h}}_{s}\right]\right) & concat \end{array}\right.$$

where $h_{t}$ and ${\bar{h}}_{s}$ are Equation (15) and Formula (16), and $\alpha_{t}$ is the score of the attention mechanism, while W and v are learnable weight parameters. It is worth mentioning that to learn common key information between pairs of trajectories, $h_{t}$ and ${\bar{h}}_{s}$ in $score\left(h_{t},{\bar{h}}_{s}\right)$ must come from different networks.

### 4.4. Output Layer

We concatenate the feature vectors from the global attention layer and perform feature fusion on positive and negative samples. After two linear layers, the results are quantified using the sigmoid function. If the result is 1, it indicates that the positive and negative samples are from the same user; if the result is 0, it means that the positive and negative samples come from different users.

### 4.5. Unknown Trajectory Information Matching

When entering an unknown trajectory, we first go to the trajectory–user library to look for trajectory candidates. The average number of candidate users can be seen in Table 1. Due to the large number of trajectories of candidate users, we sampled them. The sampling frequency defaults to 15. After sampling, the candidate trajectories of each candidate user are obtained. Then, the similarity between the unknown trajectory and the candidate trajectories is calculated. Finally, the candidate user with the highest similarity is determined as the one who generates the unknown trajectory, and this trajectory re-identification task is completed using a statistics-based method. We know that for a user, their movement trajectory is regular, and different users are unique. We can use the user movement feature to achieve our trajectory re-identification task. We employ the data from the training set to establish the trajectory–user library. Each user is linked to multiple locations, and each location point is associated with multiple users. Consequently, we record all users corresponding to each location point, thereby constituting the comprehensive trajectory–user library.

## 5. Results

In this section, we will discuss the performance of our model in real-world datasets. First, we introduce datasets, baseline models, assessment measures, and parameter settings. Then, our model is compared to the baseline model, and the impact of different strategies is analyzed. Finally, we present experimental results and model effect analysis under different constraints.

### 5.1. Experimental Settings

**Datasets.** We evaluate our approach based on three datasets, including Gowalla, Foursquare (NYC), and Weeplaces, which contain user IDs, latitude and longitude, time, and more. We preprocess the dataset, and each user’s trajectory is divided into sub-trajectories according to the sampling frequency of the day. The description of the three datasets is shown in Table 1, and we divide each dataset into two parts. By randomly sampling samples from the original dataset, the top 80% of each user is selected as the training dataset and the remaining 20% is used for testing.

**Baseline.** We compare our model with several existing approaches. The introductions of those methods are shown as follows.

- **HIST:** This method is a statistical method introduced by [38]. It is based on the classic least-cost two-part plot matching formula and realizes the re-identification task by matching the position histograms of different trajectories. It reflects the differences between different trajectories through KL divergence.
- **TULER:** TULER [15] is the first proposed deep learning model to solve the user and trajectory linking problem. First, it embeds the checked-in trajectory information into a low-dimensional vector via Word2Vec, and then encodes it through RNN neural networks (LSTM, GRU). Finally, the classifier is used to identify the user to which the track belongs.
- **TULVAE:** Zhou et al. [16] further improved the TULER network and proposed TULVAE. The network uses variational autoencoder to improve the TULER model and proposes a TULVAE model framework, which further alleviates the sparsity of position data by learning the hierarchical semantic information of the learning trajectory, and uses the semi-supervised learning method to improve the linking ability.
- **TULAR:** Ref. [17] proposed the TULAR model, which introduces trajectory semantic vectors (TSVs) through unsupervised positional representation learning and recurrent neural networks, through which partial weights of source trajectories are calculated.
- **MainTUL:** MainTUL [26] is a model for a new mutual distillation learning network to solve the TUL problem of sparse check-in moving data.
- **DeepTUL:** Miao et al. [25] proposed the DeepTUL model, which not only combines multiple characteristics of user mobility to model high-order complex mobility patterns but also learns from marked historical trajectories to capture the multi-period feature of user mobility and alleviate the problem of data sparsity.

**Parameter settings.** Table 2 lists the possible range of values of the different parameters and the values of the parameters used in our model.

**Metrics.** We used two metrics, ACC@K and Macro-f1. ACC@K is used to measure the accuracy of links. The F1 value is the harmonic mean of the precision (macro-P) and recall (macro-R). The formula is as follows:

$$ACC@K=\frac{correctly\;linked\;trajectories@K}{all\;trajectories}$$

$$Macro-f1=2\times \frac{macro-P\times macro-R}{macro-P+macro-R}$$

### 5.2. Performance Comparison

As shown in Table 3, Table 4 and Table 5, based on the three datasets, we found that our model outperformed all baselines in various evaluation indicators (Ours-GRU and Ours-LSTM are two variants of RNN encoder in our contrastive distillation model). Specifically, based on the Gowalla dataset, ACC@1, ACC@5, and Macro-F1 outperformed the second place on average by 6.14%, 6.58%, and 4.49%, respectively. Based on the Foursquare dataset, ACC@1, ACC@5, and Macro-F1 outperformed the second place on average by 25.49%, 24.76%, and 25.1%, respectively. Based on the Weeplaces dataset, ACC@1, ACC@5, and Macro-F1 outperformed the second place by an average of 18.56%, 17.91%, and 16.8%, respectively. TULER only pays attention to the relevant information between location points or a single trajectory during training and testing. Compared with TULER, TULVAE uses a generative framework to learn the potential variables of the trajectory and forms a fixed prior distribution through a variational autoencoder, but this ignores the potential distribution information of the inspected data. Although TULAR introduced the trajectory semantic vector (TSV) and attention mechanism, it still only pays attention to the correlation of multiple positions in a trajectory. Both Deep TUL and MainTUL use historical data, and DeepTUL uses historical trajectory data and current trajectory data to determine the user who belongs to the trajectory. MainTUL learns the latent information between pairs of trajectories using mutual distillation through data augmentation of historical trajectories. This means that it has higher accuracy than other methods. Different from the above approaches, our model takes into account both the position-to-position information between individual trajectories and the latent information between trajectories. The trajectory embedding of our model maps sparse trajectory points into high-dimensional spatial representations, capturing features and patterns in the trajectory data. The contrastive distillation layer learns more discriminating feature representations from the outputs of two different models, RNN and Transformer. The global attention mechanism selects and fuses the outputs from these two models to focus more on the information that contributes to our task. By combining these three modules, our model can focus not only on information at the location point level, but also on key information between trajectories. The information between trajectories pays more attention to the similarity and differences of human motion paths. As human movement patterns are regular, trajectory-level information will be more helpful for the model to mine trajectory data. In addition, we also considered making a trajectory library using historical trajectories. In our paper, we did not perform data augmentation but constructed positive and negative samples. The construction of positive and negative samples can introduce difference information between different user classes, so the model can learn more discriminating feature representations by comparing the features of positive and negative samples to better distinguish between different user categories.

We also note that for most models, model performance is worse when using data with more users than when using data with fewer users. This is intuitive because the more users there are, the more difficult it is to categorize the information. However, based on the Weeplaces dataset, the accuracy of our model does not decrease, but rather increases.

The number of users changed from 300 to 600, and our ACC@1 and Macro-F1 increased by an average of 3.09% and 3.39%, respectively. The previous model, which targeted data with fewer users, did have some improvements considering the historical data of all users. However, when the number of users is large, the large amount of historical data will also bring more noise, resulting in a sharp decrease in performance. However, our model pays attention to the correlation between pairs of trajectories during the training process and learns information between trajectories and within trajectories, so it still performs better on data with more users.

### 5.3. Ablation Study

**Figure 2:**
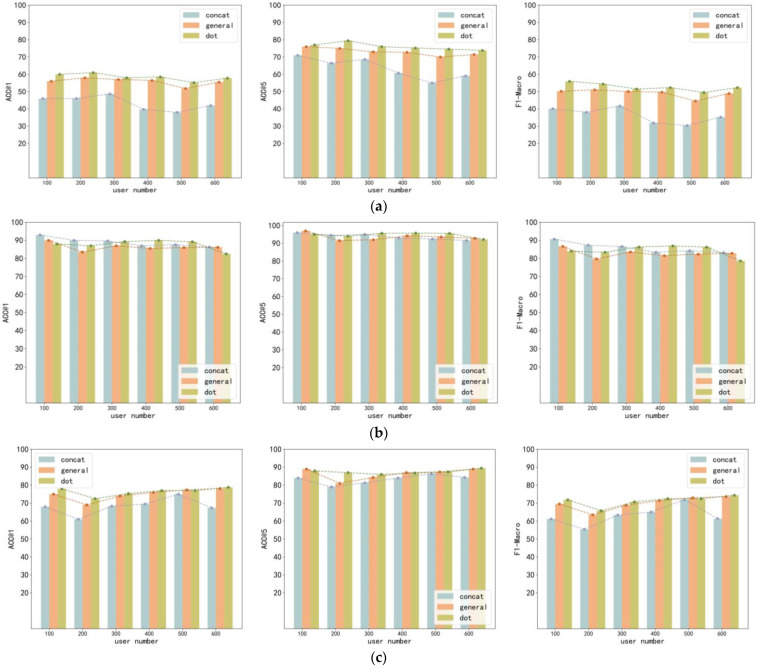
The impact of different forms of global attention mechanism on different datasets. (**a**) Gowalla. (**b**) Foursquare. (**c**) Weeplaces.

To study the impact of the three forms of global attention on our model, we selected 100–600 users from three datasets for our experiments. In Figure 2a, as the number of users increases, the performance of the model first improves and then decreases. The performance of the model is lowest when the number of users is 500. This may be due to the excessive increase in the number of users, resulting in more candidate trajectories, making it difficult for our model to correctly identify anonymous trajectories. It can also be seen from Figure 2a that the attention mechanism in the form of the dot is better than general and concat. As seen in Figure 2b, as the number of users increases, the model performance drops slightly. Based on the Foursquare dataset, the effect of the three forms of attention mechanism is not very different. As seen in Figure 2c, when the number of users is 200, the model performance reaches its lowest. Afterward, as the number of users increases, the performance of the dot and general attention mechanism models improves. However, the performance of the attention mechanism model in the form of concat drops sharply when the number of users is 600. This may be because when the number of users is 600, it is difficult for the attention mechanism model in the form of concat to distinguish trajectories in the Weeplaces dataset. Also in the Weeplaces dataset, the attention mechanism model in the form of the dot is better than general and concat.

### 5.4. Strategy Study

We evaluate the influence of different sampling frequencies of the data on the model, as can be seen in Table 6. The model with a sampling interval of one week has the lowest accuracy, which may be due to the decrease in the number and length of trajectories, making it difficult for the RNN model to learn inter-trajectory dependencies. In addition, the weekly trajectory information also includes weekend information, resulting in more noise redundancy. The spatial distribution of user check-in data is broad, and the randomness of check-in location is strong. For the Gowalla and Foursquare datasets, they are sampled at intervals of a day when there are fewer users, which is more accurate. When there are many users, sampling at 6 h intervals is more accurate.

**Figure 3:**
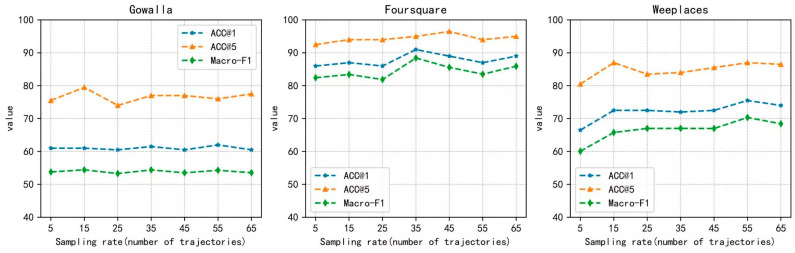
The effect of different trajectory sampling frequencies.

We also evaluate the impact of different trajectory sampling frequencies on the recognition of anonymous trajectories by our model when constructing positive and negative samples. As shown in Figure 3. For the Gowalla dataset, as the sampling frequency increases, the performance of our model does not increase, which shows that our model can cover most of the information in the Gowalla data set at a lower sampling frequency. For the Foursquare dataset, as the sampling frequency increases, the performance of the model is slightly improved, and the performance of the model reaches its best when the sampling frequency is 35. But, as the sampling frequency increases again, our model performance degrades. This may be due to the large number of candidate user trajectories, which makes it difficult for the model to find dependencies from complicated information. For the Weeplaces dataset, the sampling frequency greatly improves from 5 to 15. Afterward, as the sampling frequency increases, the performance of the model tends to be stable. This shows that our model is robust and has good generalization ability when faced with data at different sampling frequencies.

## 6. Conclusions

Given the relevant information between trajectories, a new comparative distillation network is proposed to solve the problem of trajectory re-identification. Based on the contrastive learning framework and distillation network, we design the contrastive distillation encoder layer, which introduces a global attention mechanism to allow the model to adaptively pay attention to the important information between the trajectories. Therefore, our model learns not only relevant information between location points but also information between trajectories. When we aim to identify an anonymous trajectory, we determine the candidate trajectory according to the trajectory–user library. Then, we need to calculate the similarity between the anonymous trajectory and the candidate trajectory. Finally, the similarity scores of candidate users are calculated based on statistical methods to complete the trajectory re-identification task. Our experiments on three real check-ins datasets show that our model significantly outperforms the baseline on all evaluation metrics. In future work, we will test the use of more novel datasets and increase the number of user identification.

## Figures and Tables

**Table 1:** Datasets description and statistics.

Dataset	User Number	Trajectories	POIs	Average POIs per Trajectory	Average Matcher
Gowalla	300	26,740	13,556	2.53	106.48
	600	51,714	21,161	2.65	163.27
Foursquare	300	26,473	15,772	2.33	131.44
	600	52,777	26,182	2.45	263.07
Weeplaces	300	59,138	19,607	2.67	143.54
	600	110,103	29,003	2.74	203.43

Average matcher: The average number of candidate users matched to each trajectory.

**Table 2:** Parameters used in the proposed model.

Parameters	Ours	Optional Range
POI embed dimension	250	100–300
Batch size	256	64–512
Dropout rate	0.5	0–1
Learning rate	0.01	0.001–1
Number of Transformer layer	1	≥1
Number of LSTM layers	1	≥1
Number of hidden layers	300	100–1000

**Table 3:** Performance comparison of different methods based on Gowalla.

Dataset	Methods	ACC@1	ACC@5	Macro-f1	ACC@1	ACC@5	Macro-f1
		|u| = 300			|u| = 600		
Gowalla	WYCI	40.1	55.9	33.5	41.5	56.6	35
	TULER	46.2	63.6	34.6	46.4	62	34.8
	TULVAE	46.6	64.6	37.9	46.3	62.9	35.7
	TULAR	47.8	64.9	39.8	46.6	63.1	39
	DeepTUL	48.3	65.8	40.1	45	62	34.9
	MainTUL	53.3	69.5	48.4	50.2	66.4	44.2
	Ours-GRU	59	75	52.6	56.7	73.2	50.2
	Ours-LSTM	58	76	51.5	57.8	73.8	52.3

**Table 4:** Performance comparison of different methods based on Foursquare.

Dataset	Methods	ACC@1	ACC@5	Macro-f1	ACC@1	ACC@5	Macro-f1
		|u| = 300			|u| = 600		
Foursquare	WYCI	56.05	64	55.49	53.72	63.62	52.06
	TULER	54.53	64.99	52.66	55.27	64.36	53.63
	TULVAE	53.67	64.96	51.24	55.89	65.39	53.56
	TULAR	54.18	65.13	53.34	55.64	64.68	55.78
	DeepTUL	54.23	65.88	54.35	54.36	63.17	52.37
	MainTUL	61.48	70.88	60.02	56.12	68.45	56.68
	Ours-GRU	87.67	95.67	84.44	87.65	94.17	84.43
	Ours-LSTM	89.33	95.67	86.33	82.5	92.17	78.58

**Table 5:** Performance comparison of different methods based on Weeplaces.

Dataset	Methods	ACC@1	ACC@5	Macro-f1	ACC@1	ACC@5	Macro-f1
		|u| = 300			|u| = 600		
Weeplaces	WYCI	56.59	67.77	54.13	60.88	71.34	57.65
	TULER	42.11	58.09	35.54	42.31	57.88	33.24
	TULVAE	43.16	58.64	35.86	43.25	57.67	34.12
	TULAR	44.25	58.24	37.56	44.61	57.83	36.72
	DeepTUL	40.75	54.67	33.26	38.67	49.67	30.03
	MainTUL	47.66	59.68	47.13	43.9	56.98	43.65
	Ours-GRU	76	86.67	71.31	78.67	87.67	74.37
	Ours-LSTM	75.33	86	70.68	78.83	89.5	74.4

**Table 6:** The influence of different sampling frequencies of datasets on our model.

Dataset	User Number	Sampling Rate								
		6 h			Day			Week		
		ACC@1	ACC@5	Macro-f1	ACC@1	ACC@5	Macro-f1	ACC@1	ACC@5	Macro-f1
Gowalla	|u| = 300	56.19	73.58	48.89	58	76	51.49	50.50	72.91	44.21
	|u| = 600	58.26	72.45	51.33	57.83	73.83	52.32	47.75	67.95	41.14
Foursquare	|u| = 300	89	95	86.24	89.33	95.67	86.33	65	83.67	59.70
	|u| = 600	87.17	94.5	84.02	82.5	92.17	78.58	74.46	86.31	68.57
Weeplaces	|u| = 300	78	86	73.62	75.33	86	70.68	70	86	64.79
	|u| = 600	80.17	88.33	76.08	78.83	89.5	74.4	73.5	86.33	68.42

## Data Availability

Data available in a publicly accessible repository that does not issue DOIs. Publicly available datasets were analyzed in this study. This data can be found here: https://snap.stanford.edu/data/loc-Gowalla.html, https://sites.google.com/site/yangdingqi/home/foursquare-dataset and http://www.yongliu.org/datasets/.
